# Effects of Acute Aerobic Exercise on Cognition and Constructs of Decision-Making in Adults With and Without Hypertension

**DOI:** 10.3389/fnagi.2019.00041

**Published:** 2019-03-08

**Authors:** Wesley K. Lefferts, Jacob P. DeBlois, Corey N. White, Kevin S. Heffernan

**Affiliations:** ^1^Exercise Science, Syracuse University, Syracuse, NY, United States; ^2^Department of Psychology, Missouri Western State University, St. Joseph, MO, United States

**Keywords:** acute exercise, hypertension, executive function, mathematical modeling, memory, short-term, cognition, decision making

## Abstract

Hypertension accelerates brain aging, resulting in cognitive dysfunction with advancing age. Exercise is widely recommended for adults with hypertension to attenuate cognitive dysfunction. Whether acute exercise benefits cognitive function in this at-risk population is unknown. The purpose of this study was to compare the effects of acute aerobic exercise on cognitive function in 30 middle-aged hypertensive (HTN) and 30 age, sex, and body mass index (BMI)-matched non-HTN adults (56 ± 6 years, BMI 28.2 ± 2.9 kg/m^2^; 32 men). Subjects underwent cognitive testing pre/post 30-min cycling (≈55% peak oxygen consumption). Cognition was assessed using standard metrics of accuracy and reaction time (RT) across memory recognition, 2-back, and Flanker tasks. Behavioral data was further analyzed using drift-diffusion modeling to examine underlying components of decision-making (strength of evidence, caution, bias) and RT (non-decision time). Exercise elicited similar changes in cognitive function in both HTN and non-HTN groups (*p* > 0.05). Accuracy was unaltered for Flanker and 2-back tasks, while hits and false alarms increased for memory recognition post-exercise (*p* < 0.05). Modeling results indicated changes in memory hits/false alarms were due to significant changes in stimulus bias post-exercise. RT decreased for Flanker and memory recognition tasks and was driven by reductions in post-exercise non-decision time (*p* < 0.05). Our data indicate acute exercise resulted in similar, beneficial cognitive responses in both middle-age HTN and non-HTN adults, marked by unaltered task accuracy, and accelerated RT post-exercise. Additionally, drift-diffusion modeling revealed that beneficial acceleration of cognitive processing post-exercise (RT) is driven by changes in non-decision components (encoding/motor response) rather than the decision-making process itself.

## Introduction

Cognitive function is one of the most important determinants of health, function, and quality of life with advancing age (Wilson et al., 2013). Hypertension accelerates brain aging (Gasecki et al., 2013) and impairs cognitive function (Elias et al., 1993; Shehab and Abdulle, 2011; Iadecola et al., 2016). The cognitive domains of executive function [high-level interrelated cognitive abilities that integrate lower-level functions to complete goal-directed behavior (Logue and Gould, 2014)] and memory appear to be most vulnerable to hypertension (Shehab and Abdulle, 2011; Hughes and Sink, 2015). Identifying lifestyle modifications that can improve cognitive function in these domains is therefore of particular interest for adults with hypertension.

Aerobic exercise is highly recommended (Pescatello et al., 2004; Brook et al., 2013) for adults with hypertension to not only help control blood pressure but to maintain cognitive health (Gorelick et al., 2011) and prevent cognitive impairment with advancing age (Pedersen and Saltin, 2006; Lange-Asschenfeldt and Kojda, 2008). Despite these recommendations there is a paucity of data on the effect of exercise on cognitive function in adults *with hypertension*. Indeed, only 1 study has investigated the effects of exercise training on cognitive function in hypertension (noting no significant effects) (Teixeira et al., 2015). Previous studies have suggested the acute cognitive response to exercise may offer insight into origins of neuro-cognitive training adaptations (Basso and Suzuki, 2017). Cognitive responses to acute exercise may additionally provide insight into underlying neural plasticity by revealing whether exercise induces acute changes in cognitive function (Ludyga et al., 2016). Hypertension is linked to diffuse structural brain damage/atrophy (Gasecki et al., 2013) which may disrupt neural plasticity and alter the ability of the brain to respond to exercise. Currently, however, no data exists on acute cognitive responses to exercise in hypertension.

Meta-analytical investigations in non-hypertensive populations have shown that acute exercise improves executive function (Tomporowski, 2003; Chang et al., 2012; McMorris and Hale, 2012) and may positively impact memory (Tomporowski, 2003; Chang et al., 2012). Moderate to vigorous exercise appears to produce the most pronounced, beneficial changes in both executive function, and memory (Tsukamoto et al., 2017). Whether hypertensives experience similar improvements in executive function and memory performance as their non-hypertensive counterparts following a bout of exercise is unknown.

When studying the effects of exercise on cognitive function, traditional analytical approaches rely on reverse inference to survey accuracy or processing speed/reaction time (RT). Greater accuracy is believed to reflect better functioning of the cognitive domain of interest (i.e., executive function, memory). Faster RT is also posited to indicate improved functioning in the cognitive domain of interest since less time is required for the individual to respond correctly to the stimulus. Reverse inference is valid only if the changes in behavior (in this example RT) are driven solely by the cognitive domain being surveyed. There is substantial evidence that factors related to the decision making process impact both RT and accuracy separate from any direct impact on the cognitive domain of interest (White et al., 2016).

Drift-diffusion modeling (DDM) is a mathematical approach (Forstmann and Wagenmakers, 2015) that decomposes observational data into latent processes underlying decision-making. Constructs elucidated by DDM include caution, encoding, motor response duration, strength, and quality of evidence presented by the stimulus, and bias (i.e., implicit or explicit preference for one response over another) (White et al., 2011, 2016; White and Poldrack, 2014). Thus, DDM attempts to describe changes in the latent decision-making process that are responsible for the observed responses. DDM incorporates *all available behavioral data* (accuracy, correct/error RT, shape of RT distributions) rather than solely relying on RT for correct trials and accuracy to describe changes in behavior. This modeling technique can provide novel insight into whether changes in cognitive function stemming from acute exercise (observed through accuracy and RT) are due to neural (i.e., encoding, motor response) or behavioral changes (i.e., caution, bias) in the latent constructs of decision-making.

As such, the purpose of this investigation was to examine the effect of acute aerobic exercise on cognitive function (using memory and executive function tasks) and latent constructs of decision making in hypertensive (HTN) and non-hypertensive (Non-HTN) middle-aged adults. It was hypothesized that acute exercise would differentially affect cognitive function in HTN (unaltered performance) and Non-HTN (improved performance; manifesting as improved accuracy and accelerated RT post-exercise on executive function and memory tasks).

## Materials and Methods

### Participants

Thirty middle-aged HTN (56 ± 6 years; 14 women) and 30 age-, sex-, and body mass index (BMI)-matched non-HTN adults (56 ± 6 years; 14 women) were recruited for this study. This investigation was part of a larger study designed to investigate the vascular and cognitive responses to acute exercise in hypertension. While the vascular responses are published elsewhere (Lefferts et al., 2018b) the current paper will present the cognitive results. We targeted middle-aged adults because (1) cognitive decline can be detected as early as middle-age (Singh-Manoux et al., 2012), making this age range a prime target for preventive research and (2) recent meta-analyses indicate this is an understudied group regarding acute exercise and cognitive function (Ludyga et al., 2016). Exclusion criteria included self-reported smoking, stroke, dementia, diabetes mellitus, severe obesity (BMI ≥ 35 kg/m^2^), previous cardiovascular events, pulmonary/renal/neurological disease, or recent head trauma (concussion). Participants were free from dementia (Montreal Cognitive assessment score ≤ 21), and depression [assessed using the center for epidemiologic studies depression (CESD) questionnaire]. Hyperlipidemic and overweight participants (BMI 25–30 kg/m^2^), were included in the sample due to the high prevalence of these risk factors within middle-aged adults (regardless of HTN status). Menopausal status (pre-, peri-, post-menopausal) was assessed according to STRAW+10 guidelines. This study was carried out in accordance with the recommendations of Syracuse University Institutional Review Board. The protocol was approved by the Syracuse University Institutional Review Board. All subjects gave written informed consent in accordance with the Declaration of Helsinki prior to study initiation.

All HTN participants were diagnosed and undergoing treatment for hypertension. Participants did not refrain from HTN medication during testing due to concern of rebound hypertension, and to improve external validity, as the medicated state is the “natural state” in which the majority of HTN would engage in exercise. All participants underwent descriptive testing (fasting lipid/glucose assessment, anthropometrics, body composition, VO_2_peak assessment during an incremental cycling protocol to volitional fatigue) and familiarization prior to the acute exercise visit. For more information on these methods, the reader is directed to the following reference (Lefferts et al., 2018b).

### Study Design

**Figure 1:**
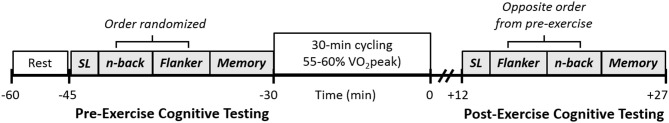
Study design for assessing cognitive function pre and post moderate intensity exercise aerobic exercise. SL, study list.

Participants were instructed to arrive >4-h fasted, and abstain from non-essential medication, caffeine, alcohol, and exercise the day of the visit. All acute exercise visits were standardized to the morning. Participants underwent cognitive measures pre and post a 30-min bout of aerobic exercise (Figure 1). Pre-exercise cognitive testing occurred following 15-min of supine rest. Post-exercise measures were assessed ~10–12 min post because cognitive function may be negatively affected within the first 10 min post exercise (Chang et al., 2012). All pre and post measures were assessed in the supine position.

### Acute Exercise Protocol

The acute aerobic exercise bout was 30-min of moderate-intensity cycling (57.1 ± 3.5% VO_2_peak). This exercise dose/intensity is recommended by the American Heart Association and American College of Sports Medicine for adults <65 years of age (Haskell et al., 2007), and elicits positive effects on cognitive function post-exercise (Chang et al., 2012) in healthy adults. Exercise intensity was confirmed and titrated by measuring oxygen consumption during two separate periods of the exercise bout (min 5–10, and 20–25).

#### Cognitive Measures

Cognitive function was assessed using a 15-min, 3-task computerized (Matlab, The MathWorks, Natick, MA; and PsychToolbox) cognitive battery (with a hand-held response clicker) that interrogates memory (word recognition task) and the attention (Flanker task), and working memory (2-back version of an n-back task) components of executive function. This battery of tasks has been used by our group previously (Lefferts et al., 2016). Prior to starting the current study, we tested the within- (tests separated by 5-min break) and between-day (24-h between tests) reliability of our cognitive tasks in 19 healthy adults (age range 18–35 yr; 8 women). This data indicated moderate to strong within-day reliability (ICC's 0.62–0.80) for cognitive metrics (hits/reaction time) for memory recognition and Flanker tasks (see Supplemental Table S1). Within-day reliability of the 2-back was low from this preliminary study, so 2-back task rules were adjusted prior to conducting the current study such that participants were required to respond to non-match items (rather than solely match items). Literature suggests the memory recognition paradigm and flanker task are valid and reliable measures of memory and attention, respectively (Fuchs et al., 1999; Zelazo et al., 2014). While there are mixed findings regarding the validity of the 2-back as a measure of working memory, it is reliable (Jacola et al., 2014) and helpful in identifying differences in cognitive processing between groups/clinical populations (Miller et al., 2009), and following acute exercise (Basso and Suzuki, 2017). These particular domains were selected because they have implications for later-life cognitive function and are affected by HTN and acute exercise (Chang et al., 2012). The battery always began with the word study list, followed by the working memory and executive function tasks in a randomized, counter-balanced order, followed by the memory recognition task. Each trial was preceded by verbal instructions and a visual reminder of each task and its respective goals, in addition to brief on-screen instructions prior to beginning each task once testing had begun. This multi-stage instruction process was designed to ensure participants recalled the goal of each task prior to initiation.

Participants were familiarized with all cognitive tasks prior to the acute exercise visit to account for learning effects. Familiarization included point-by-point written and verbal instructions for each task, followed by a complete practice session of the cognitive battery. If participants did not adequately understand a task they were permitted to repeat the task until they were comfortable with the goals and procedures of the task.

### Memory

Memory recognition was assessed by presenting participants with 36 words for memorization and later recognition from memory. The list contained 36 concrete words from the English language that were displayed for 1 s each. Participants then completed two cognitive tasks (flanker and 2-back, randomized order) before beginning the memory recognition portion of the test (~10 min later). To assess memory recognition performance, participants were presented with 72 words (36 distracters), at a rate of 1 every 2 s, and instructed to identify the words as “old” if they remembered the word from the study list or “new” if the word being presented was not on the study list. Accuracy was expressed as percent hits (correctly recalled items) and false alarms (old/studied items incorrectly identified as new/distractors). Mean reaction time (RT) for hits was calculated to assess processing speed.

### Executive Function

The attention component of executive function was assessed using the Eriksen Flanker task. Participants were presented with 5 arrows (standard Eriksen Flanker task) and instructed to respond to which direction the middle arrow was pointing. This task contained 64 congruent (i.e., all arrows facing the same direction; <<<<<) and 64 incongruent (i.e., flanking arrows facing different direction from middle arrow; <<><<) 1.5-s long trials (totaling ~5-min). The 128 items were accumulated through 2 consecutive runs of 64 congruent/incongruent items, separated by a 10-s break. Accuracy was expressed as percent hits and calculated as hits / (hits + incorrect), where missed stimuli (i.e., no response within the 1.5 s window) were ignored and not counted as incorrect. Processing speed was assessed as mean hit RT for hit incongruent/congruent trials.

The working memory component of executive function was assessed using a 2-back number task. This task included 160 trials divided into 2 runs of 80 items, separated by a 10-s break. The task lasted ~4 min. After fixating on 3 crosses, participants were presented with a series of digits (1–9) at a rate of 1/s, with consecutive numbers separated by 0.25 s. They were instructed to press the right response button if the number presented matched the number that was presented 2 numbers before (i.e., 4-7-4) and press the left response button for all non-match stimuli. Accuracy was expressed as percent hits (hits/(hits + errors)) and commission errors (falsely identifying a number as a match), and processing speed was assessed as mean hit RT.

### Drift-Diffusion Modeling

Drift-diffusion modeling (DDM) was conducted *post-hoc* on all cognitive performance data. This modeling technique can provide insight into whether changes in cognitive function are due to neurological (i.e., encoding) or behavioral (i.e., caution, bias) changes. DDM has been validated (Voss et al., 2004) and described in detail previously (White et al., 2011; White and Poldrack, 2014). In short, the model assumes that decisions start at a point (*z*) and noisy evidence is sampled until a boundary (a/0) is reached, initiating a response. Wider distances between boundaries (a) indicate slower but more accurate responses (caution). Drift rate (*v*) indicates the strength of evidence (higher drift rate means stronger evidence), and non-decision time estimates the duration of encoding/motor response. DDM parameters are visually represented and summarized in our previous work (Lefferts et al., 2016).

#### Statistical Analyses

No studies to date have examined the acute effects of exercise on cognitive function adults with HTN compared to Non-HTN, making it difficult to directly power this study to detect group-by-time interactions. Previous pilot data in young adults indicated changes in RT had an effect size (Cohen's d) of 0.82 for reductions in post-exercise RT. *A priori* power calculations (2-tailed *t*-test, independent groups) using the effect size of 0.82, a power of 0.80, and α of 0.05 indicated 25 participants per group would be sufficient to detect changes in cognitive function post-exercise. This sample size is similar to studies examining effects of blood pressure on cognitive function (≈25 per group) (Shehab and Abdulle, 2011). As such, we collected data on 30 HTN and 30 Non-HTN adults.

All data is reported as mean ± standard deviation and statistical significance was established *a priori* as *p* < 0.05. Data normality was assessed quantitatively using the Shapiro-Wilk test. Between-day and within-day reliability were analyzed using intraclass correlation coefficients from the cognitive familiarization trial and pre-exercise cognitive testing (run 1 vs. run 2 of executive function tasks). Non-normally distributed RT and DDM metrics were transformed to meet normality assumptions. Descriptive characteristics were compared using independent *T*-tests for continuous variables and χ^2^ tests for categorical data. We examined cognitive RT and DDM parameters in non-HTN vs. HTN groups across pre- and post-exercise time points using a 2 x 2 [2 group x 2 time] repeated measures ANOVA. If a significant group x time interaction was detected, it was further explored using Bonferroni corrected *post-hoc* tests. All accuracy metrics (hit rates) were unable to be successfully transformed to meet assumptions. Accuracy metrics were therefore analyzed using Mann-Whitney *U*-tests to test the effect of group (HTN vs. Non-HTN), and group by time interaction (change in accuracy post-pre for HTN vs. Non-HTN), with Wilcoxon signed-rank tests used to test the effect of time (pre- vs. post-exercise). All significant non-parametric analyses were corrected for multiple comparisons via Bonferroni correction since these analyses could not be run simultaneously. Effect sizes are presented with their corresponding *p*-values and were calculated as Cohen's D, partial eta squared (η^2^), and r^2^ (Z^2^/n) for descriptive characteristics, ANOVA, and non-parametric analyses, respectively.

## Results

### Cognitive Task Reliability

Intraclass correlation coefficients for between-day reliability were statistically significant and ranged from moderate to strong across all cognitive tasks (Supplemental Table S1). Pertinent to this study design, we noted moderate to strong within-day reliability for the pre-exercise Flanker and 2-back tasks (intraclass correlation coefficients 0.72–0.92, *p* < 0.05).

### Group Characteristics and Acute Exercise Intensity

As described elsewhere (Lefferts et al., 2018b), groups were well-matched for descriptive characteristics (including sex, age, body size, body composition, fasting lipids, depression symptomology, and menstrual status; Table 1). Fasting glucose was significantly higher in HTN vs. Non-HTN (*p* < 0.05). Cardiorespiratory fitness and accumulated minutes of moderate-to-vigorous physical activity (*p* < 0.05) were significantly higher, and average number of steps over 6 d tended to be higher in Non-HTN (*p* = 0.06) compared to HTN. Statin use was greater in HTN vs. Non-HTN (*p* < 0.05). On average, HTN participants had been diagnosed for 129 ± 97 months. HTN participants took their blood pressure medication at similar times of day (63.3%, AM vs. 33.0%, PM; *p* = 0.095), with one participant taking medication at both times of day. At-home systolic and diastolic blood pressure were higher in HTN (systolic 126 ± 12 mmHg, diastolic 79 ± 8 mmHg) than Non-HTN (systolic 116 ± 9 mmHg, diastolic 73 ± 6 mmHg; *p* < 0.05).

**Table 1:** Descriptive characteristics for non-HTN and HTN groups (mean ± SD unless otherwise noted).

	**Non-HTN**	**HTN**	***p*-Value (effect size)**
Sex (male/female)	16/14	16/14	–
Age (years)	56 ± 6	56 ± 6	0.93 (0.00)
**ANTHROPOMETRICS**			
Height (cm)	169.8 ± 11.3	171.3 ± 9.6	0.57 (0.14)
Weight (kg)	82.0 ± 13.3	82.4 ± 12.5	0.91 (0.03)
Body fat (%)	32.2 ± 8.4	31.4 ± 6.9	0.69 (0.10)
Body mass index (kg/m^2^)	28.3 ± 2.6	28.0 ± 3.3	0.71 (0.10)
**MEDICATIONS, %(*****n*****)**			
Statin	6.7 (2)	40.0 (12)	**0.01**
Birth control	3.3 (1)	0.0 (0)	–
Hormone replacement therapy	3.3 (1)	3.3 (1)	–
Hypothyroid	3.3 (1)	3.3 (1)	–
ACE inhibitor	–	43.3 (13)	–
ARB	–	33.3 (10)	–
Diuretic	–	50.0 (15)	–
β-Blocker	–	13.3 (4)	–
CCB	–	13.3 (4)	–
Combination therapy (≥2)	–	43.3 (13)	
**LIPID PROFILE**			
Hemoglobin (g/dL)	14.2 ± 0.9	13.8 ± 1.3	0.12 (0.36)
Total cholesterol (mg/dL)	202 ± 39	192 ± 36	0.28 (0.27)
HDL (mg/dL)	58 ± 17	56 ± 20	0.56 (0.11)
Triglycerides (mg/dL)	103 ± 61	116 ± 56	0.28 (0.22)
LDL (mg/dL)	128 ± 41	114 ± 30	0.17 (0.39)
Non-HDL (mg/dL)	144 ± 44	136 ± 32	0.43 (0.21)
Total cholesterol:HDL	4 ± 2	4 ± 1	0.89 (0.00)
Glucose (mg/dL)	94 ± 9	102 ± 16	**0.03 (0.62)**
**QUESTIONNAIRES**			
CESD	6 ± 5	7 ± 4	0.71 (0.22)
**FITNESS**			
VO_2_peak (mL/kg/min)	32.4 ± 8.8	27.2 ± 5.6	**0.01 (0.71)**

*ACE, angiotensin converting enzyme; ARB, angiotensin II receptor blocker; CCB, calcium-channel blocker; HDL, high-density lipoprotein; LDL, low-density lipoprotein; CESD, Center for Epidemiologic Studies Depression questionnaire; VO_2_peak, peak oxygen consumption. Effect size calculated as Cohen's D. Bold value denotes p < 0.05*.

### Acute Exercise

There were no differences in aerobic exercise intensity between HTN and Non-HTN for %relative VO_2_peak (Non-HTN, 57.6 ± 3.6% vs. HTN, 56.5 ± 3.5% *p* > 0.05) and %maximal heart rate (Non-HTN, 69.7 ± 5.5 vs. HTN, 71.4 ± 7.3 *p* > 0.05). Absolute workload during exercise was higher for Non-HTN vs. HTN (Non-HTN, 82 ± 43W vs. HTN, 63 ± 22W *p* < 0.05).

### Post-exercise Testing

Participants began supine rest within 27 ± 6 and 26 ± 6 s following cessation from exercise for Non-HTN and HTN groups, respectively (*p* > 0.05). Post-exercise cognitive testing was initiated at similar times between groups (memory study list, Non-HTN, 12.12 ± 1.25 min vs. HTN, 11.96 ± 1.10; Flanker, Non-HTN, 16.14 ± 2.67 min vs. HTN, 16.78 ± 4.00 min; 2-back, Non-HTN, 17.05 ± 3.26 min vs. HTN 16.74 ± 3.4 min; Memory, Non-HTN, 24.27 ± 1.23 min vs. HTN 23.57 ± 2.34 min; *p* > 0.05). The higher variability for post-exercise timing of Flanker and 2-back is related to the randomized, counter-balance design.

### Effect of Exercise on Accuracy and RT

Hand-held clicker malfunctions resulted in lost data for 2 non-HTN individuals on the Flanker task and 1 non-HTN individual on the 2-back. Thus, data are presented for *n* = 28 and 29 for Flanker and 2-back, respectively, among Non-HTN. No significant group, time, or group-by-time effects were detected for accuracy as measured by hit rates on congruent/incongruent Flanker or 2-back (*p* > 0.05; Table 2). A time effect was detected for memory hit rate, which increased post-exercise in both groups (*p* < 0.05; Table 3). This was accompanied by a time effect for false alarm rate, which also increased post-exercise (*p* < 0.05). Time effects were detected for Flanker and Memory hit RT, which decreased post-exercise (*p* < 0.05). There was a tendency for hit RT to decrease post-exercise on the 2-back although this did not reach statistical significance (*p* = 0.09). No statistical effects for discriminability were detected. No group or group by time interactions were observed at the *p* < 0.05 level, indicating that (1) there were no inherent statistical group differences in cognitive performance and (2) the statistical effect of acute exercise on accuracy and processing speed were similar between HTN and Non-HTN individuals.

**Table 2:** Executive function parameters pre and post-exercise in HTN and Non-HTN individuals (mean ± SD).

**Parameter**	**Non-HTN**		**HTN**		***p*****-Value (effect size**, **η**^**2**^**)**		
	**Pre**	**Post**	**Pre**	**Post**	**Group**	**Time**	**GxT**
**FLANKER**							
Hits, congruent (%)^†^	99.9 ± 0.3	99.8 ± 0.7	99.9 ± 0.6	99.8 ± 0.6	0.93 (0.00)	0.58 (0.01)	0.94 (0.00)
Hit RT, congruent (ms)	513 ± 76	493 ± 66	510 ± 64	478 ± 63	0.63 (0.00)	**0.01 (0.35)**	0.15 (0.04)
Hits, incongruent (%)^†^	97.8 ± 3.0	98.0 ± 2.5	96.8 ± 4.5	95.5 ± 4.9	0.54 (0.01)	0.80 (0.00)	0.83 (0.00)
Hit RT, incongruent (ms)	606 ± 87	578 ± 82	590 ± 68	562 ± 72	0.46 (0.06)	**0.01 (0.34)**	0.89 (0.00)
*Caution*	0.22 ± 0.14	0.21 ± 0.12	0.24 ± 0.24	0.24 ± 0.20	0.77 (0.00)	0.97 (0.00)	0.80 (0.00)
*Non-decision time (ms)*	309 ± 121	296 ± 117	320 ± 83	273 ± 112	0.81 (0.00)	0.07 (0.06)	0.28 (0.02)
*Attention interference (ms)*	190 ± 171	152 ± 103	198 ± 176	158 ± 108	0.55 (0.01)	0.26 (0.02)	0.78 (0.00)
*Perceptual strength*	0.53 ± 0.19	0.55 ± 0.17	0.58 ± 0.19	0.59 ± 0.18	0.26 (0.02)	0.35 (0.02)	0.69 (0.00)
**2-BACK**							
Hits (%)^†^	67.0 ± 22.2	67.2 ± 21.5	73.5 ± 15.6	74.1 ± 17.0	0.26 (0.02)	0.96 (0.00)	0.69 (0.00)
Commission errors (%)^†^	6.4 ± 4.9	5.6 ± 5.2	7.3 ± 6.6	6.2 ± 5.5	0.67 (0.00)	0.07 (0.13)	0.98 (0.00)
Discriminability^†^	0.61 ± 0.22	0.62 ± 0.20	0.66 ± 0.15	0.679 ± 0.168	0.33 (0.02)	0.76 (0.00)	0.71 (0.00)
Hit RT (ms)	666 ± 106	651 ± 94	633 ± 89	613 ± 88	0.13 (0.04)	0.09 (0.05)	0.66 (0.00)
*Caution*	0.14 ± 0.02	0.15 ± 0.02	0.15 ± 0.02	0.14 ± 0.02	0.44 (0.01)	0.28 (0.02)	0.06 (0.06)
*Non-decision time (ms)*	402 ± 108	370 ± 95	353 ± 94	344 ± 91	0.11 (0.04)	**0.048 (0.07)**	0.27 (0.02)
*Response bias*	0.39 ± 0.11	0.39 ± 0.12	0.40 ± 0.11	0.39 ± 0.10	0.66 (0.00)	0.53 (0.01)	0.52 (0.01)
*Drift rate-match*	0.16 ± 0.03	0.17 ± 0.04	0.17 ± 0.04	0.17 ± 0.03	0.23 (0.03)	0.84 (0.00)	0.94 (0.00)
*Drift rate-non-match*	−0.13 ± 0.04	−0.13 ± 0.03	−0.13 ± 0.04	−0.14 ± 0.03	0.65 (0.00)	0.36 (0.02)	0.61 (0.01)
*Drift rate-discriminability*	0.29 ± 0.06	0.30 ± 0.04	0.31 ± 0.05	0.31 ± 0.03	0.20 (0.03)	0.43 (0.01)	0.90 (0.00)
*Stimulus bias*	0.03 ± 0.05	0.03 ± 0.06	0.04 ± 0.60	0.03 ± 0.04	0.70 (0.00)	0.62 (0.00)	0.56 (0.01)

*Italics, Drift-diffusion modeling parameter; HTN, Hypertensive; GxT, Group-by-time interaction; RT, reaction time*.

† *Non-parametric effect size, Z^2^/n. Bold value denotes p < 0.05*.

**Table 3:** Memory recognition parameters pre and post-exercise in HTN and Non-HTN individuals (mean ± SD).

**Parameter**	**Non-HTN**		**HTN**		***p-*****Value (effect size**, **η^2^)**		
	**Pre**	**Post**	**Pre**	**Post**	**Group**	**Time**	**GxT**
**MEMORY**							
Hits, studied (%)^†^	51.8 ± 21.0	57.7 ± 20.2	53.5 ± 17.7	57.8 ± 20.7	0.71 (0.00)	**0.02 (0.12)**	0.85 (0.00)
False alarms (%)^†^	22.6 ± 17.7	27.2 ± 19.9	24.6 ± 17.7	30.2 ± 20.9	0.64 (0.00)	**0.01 (0.17)**	0.88 (0.00)
Discriminability	0.29 ± 0.18	0.30 ± 0.14	0.29 ± 0.16	0.28 ± 0.19	0.65 (0.00)	0.92 (0.00)	0.57 (0.01)
Hit RT (ms)	941 ± 181	897 ± 148	894 ± 149	853 ± 145	0.24 (0.03)	**0.02 (0.10)**	0.91 (0.00)
*Caution*	0.14 ± 0.02	0.13 ± 0.03	0.12 ± 0.02	0.13 ± 0.02	0.27 (0.02)	0.74 (0.00)	0.07 (0.05)
*Non-decision time (ms)*	579 ± 126	531 ± 104	564 ± 101	532 ± 136	0.79 (0.00)	**0.01 (0.14)**	0.53 (0.01)
*Response bias*	0.06 ± 0.02	0.07 ± 0.02	0.06 ± 0.01	0.06 ± 0.02	0.45 (0.01)	0.93 (0.00)	0.68 (0.00)
*Drift rate-studied*	0.01 ± 0.07	0.04 ± 0.07	0.02 ± 0.06	0.02 ± 0.11	0.95 (0.00)	0.12 (0.04)	0.26 (0.02)
*Drift rate-distractor*	−0.11 ± 0.08	−0.09 ± 0.07	−0.10 ± 0.09	−0.08 ± 0.10	0.75 (0.00)	0.11 (0.04)	0.87 (0.00)
*Drift rate-discriminability*	0.11 ± 0.08	0.12 ± 0.06	0.11 ± 0.07	0.10 ± 0.07	0.66 (0.00)	0.94 (0.00)	0.24 (0.02)
*Stimulus bias*	−0.10 ± 0.12	−0.05 ± 0.12	−0.08 ± 0.133	0.04 ± 0.14	0.44 (0.01)	**0.02 (0.10)**	0.76 (0.00)

*Italics, Drift-diffusion modeling parameter; HTN, Hypertensive; GxT, Group-by-time interaction; RT, reaction time*.

† *Non-parametric effect size, Z^2^/n. Bold value denotes p < 0.05*.

### Effect of Exercise on Constructs of Decision-Making

Significant time effects were detected for memory stimulus bias (increased post-exercise), and 2-back and memory non-decision time (decreased post-exercise). Similar effects were noted for the Flanker non-decision time post-exercise but this did not reach statistical significance (*p* = 0.066). No significant group or group by time interactions were detected for DDM metrics, indicating that (1) there were no underlying statistical group differences in decision making, and (2) the statistical effect of acute exercise on latent processes of decision making were similar between HTN and Non-HTN groups.

## Discussion

Acute exercise did not alter accuracy, but increased processing speed (decreased RT), on executive function and memory tasks post-exercise in both HTN and non-HTN individuals. Our DDM data indicate that non-decision time significantly decreased post-exercise during executive function and memory tasks, and that memory stimulus bias increased post-exercise. Our data cumulatively suggest that middle-age HTN experience similar beneficial increases in executive function and memory processing speed following acute exercise as their non-HTN counterparts, and that post-exercise increases in processing speed are driven by changes outside of the decision-making process.

### Hypertension and Cognitive Function

We noted no group differences in cognitive function at the *p* < 0.05 level between HTN and non-HTN in our cohort of middle-aged adults. Indeed, task accuracy and processing speeds (RT) were similar across executive function (Flanker, 2-back) and memory recognition tasks between groups. The similar executive function and memory performance between HTN and non-HTN groups contrasts with previous data (Elias et al., 1993; Li et al., 2017). These conflicting data may relate to our middle-aged sample of HTN since age independently contributes to cognitive function among hypertension (Muela et al., 2017). As such, younger HTN may not experience notable differences in cognitive function since the duration the brain is exposed to hypertension likely contributes to the degree of dysfunction (or lack thereof) (Elias et al., 1993). This suggests that our cohort of middle-aged HTN had similar cognitive health as their non-HTN counterparts, and were not currently exhibiting signs of cognitive dysfunction from accelerated brain aging. Ultimately, similar levels of brain health in adults with and without HTN may have preserved brain plasticity among HTN individuals and contributed to similar cognitive responses to acute exercise observed herein.

Our HTN cohort partially relied on anti-HTN medication to control their blood pressure to within normal levels which may attenuate differences in cognitive function between our groups (Muela et al., 2017). Whether anti-HTN therapy independently impacts cognitive function is of debate (Iadecola et al., 2016) and may depend on age, drug type, and cognitive assessment strategy (Gasecki et al., 2013; Kherada et al., 2015). The American Heart Association and American Stroke Association have recommended anti-HTN therapy for adults in mid-life and early old age as an effective means to attenuate late-life dementia (Gorelick et al., 2011). Additionally, some drug types (angiotensin II receptor blockers [ARB], angiotensin converting enzyme inhibitors [ACE-I], and calcium-channel blockers [CCB]) and combination therapies appear more effective in combating cognitive decline in HTN than other monotherapy drugs (Kherada et al., 2015). Half of our hypertensive cohort were on a combination therapy (i.e., >1 anti-hypertensive drug) and 90% used either an ARB, ACE-I, or CCB, which may have contributed to the similar cognitive health between our HTN and non-HTN groups. Taken together, these data indicate that our sample of medicated, well-controlled, middle-aged HTN had similar cognitive function as their non-HTN counterparts, potentially contributing to similar cognitive responses to acute exercise.

### Hypertension, Exercise, and Cognitive Function

To our knowledge, this is the first study to investigate the acute effects of exercise on cognitive function in adults with hypertension, a population at-risk for cognitive decline. Accuracy on executive function tasks (Flanker and 2-back) was unaltered by acute exercise in both HTN and non-HTN groups at the *p* < 0.05 level. We did note, however, a significant increase in memory recognition hits and false alarms post-exercise in both HTN and Non-HTN individuals. Although the percentage of correctly identified “studied” words (i.e., hits) increased post-exercise, this is not indicative of improved memory recognition performance or accuracy *per se* since it was accompanied by an increase in the percentage of false alarms (incorrect responses where distractor words were classified as “studied”). This seems to suggest that acute exercise altered how individuals categorized memory stimuli and will be discussed further below with insight from DDM.

The small effects of acute exercise on cognitive task accuracy observed herein concurs with previous experimental data (Tsai et al., 2014; Ji et al., 2017; Tsukamoto et al., 2017). Meta-analytical investigations suggest there may be a small effect on non-time dependent cognitive task performance (i.e., accuracy) (Ludyga et al., 2016), although this is not universal (McMorris and Hale, 2012). Improvements in cognitive task accuracy post-exercise may be difficult to observe as many studies rely on tasks vulnerable to ceiling effects (i.e., task difficulty is not sufficient that improvements in cognitive processing or decision-making can alter hit rates). Indeed, this may impact our findings with the Flanker task (hit rate ≈ 99%), although ceiling effects likely did not impact the effect of exercise on 2-back or memory recognition tasks based on the lower mean hit rates (≈70 and 53%, respectively). These data indicate that acute aerobic exercise does not statistically improve accuracy on executive function or memory tasks in middle-aged adults with and without hypertension.

We noted faster executive function and memory processing speed post-exercise in both adults with and without hypertension. Accelerated processing speed post-exercise manifested as significantly reduced RT for Flanker/memory recognition tasks, with smaller effects (statistical trends) for 2-back. This facilitation of RT is in-line with recent literature (Tsai et al., 2014; Ji et al., 2017; Tsukamoto et al., 2017) as well as meta-analyses of the literature at-large that indicate RT is sensitive to changes with acute exercise (Tomporowski, 2003; Ludyga et al., 2016). Previous findings suggest larger benefits of exercise on RT are seen in children and older adults, with attenuated benefits in young healthy adults (Ludyga et al., 2016). While the effect of acute exercise on cognitive function is under-investigated among middle-aged adults (Ludyga et al., 2016), our observation of accelerated RT post-exercise suggests middle-aged adults exhibit similar facilitation of post-exercise RT as children and older adults. These data suggest that middle-aged, medicated HTN experience similar facilitation of cognitive processing speed (reduced RT), on executive function, and memory tasks as their non-HTN counterparts following acute exercise.

Contrary to our hypothesis, exercise elicited similar beneficial effects on cognitive function (faster cognitive processing speeds) in HTN and Non-HTN adults. Similar cognitive responses to acute exercise in HTN and Non-HTN adults may relate to similar vascular health. Vascular health has been directly linked to both brain health and cognitive function (Gorelick et al., 2011). Our HTN cohort utilized both medication (anti-HTN, statins) and physical activity to achieve adequate blood pressure control (Lefferts et al., 2018a,b). This combined approach may have had favorable effects on their vascular health (Gepner et al., 2017). Indeed, our sample of HTN had similar vascular stiffness and cerebrovascular responses to cognitive activity as their Non-HTN counterparts (Lefferts et al., 2018a). Moreover, acute exercise had similar effects on the cerebrovasculature in our HTN and Non-HTN adults (Lefferts et al., 2018b). As such, brain health and plasticity may have been partially supported in this sample of HTN through vascular health, ultimately giving way to similar cognitive responses to exercise.

### Exercise, Cognitive Function, and Insight From Drift-Diffusion Modeling

Previous studies have provided limited insight into psychological factors underlying exercise-induced changes in cognitive function. A reason for this may be due to reliance on standard metrics of cognitive performance (task accuracy and RT) (Ludyga et al., 2016). Any change in cognitive function could stem from multiple components of the decision-making process that ultimately influence RT. Mathematical modeling via DDM is a novel means of dissecting the entire decision-making process contained in the behavioral data into its underlying components of visual encoding, evidence accumulation and decision, and motor response. As such, DDM may offer insight into mechanisms behind changes in cognitive performance (hits and false alarms) and mechanisms of accelerated RT following exercise by quantifying the changes in the decision-making process that elicited the observed responses.

We noted no statistically significant effect of acute exercise on executive function task accuracy, as assessed by hit rate on the Flanker and 2-back. The ability to correctly identify stimuli in a given task is strongly dependent on the strength of evidence extracted from the stimuli itself (White et al., 2016). An increase in the strength of evidence extracted from the stimuli would be expected to increase the hit rate and potentially decrease RT (stronger evidence reaches decision boundary faster). Since Flanker and 2-back hit rates were statistically unaltered by exercise, it is not surprising that the strength of evidence (drift rate) did not change post-exercise for either executive function task.

We did, however, observe increases in hit and false alarm rates during the memory recognition task post-exercise. This effect of exercise on task performance reflects a change in how evidence was extracted from the stimuli (quantified as drift rate by DDM). Indeed, DDM revealed that drift rates for both old/studied and new/distractor words increased (non-significantly) post-exercise. According to the model, greater absolute drift rates indicate stronger perceived evidence for a given stimuli (positive drift rates for “old/studied,” and negative for “new/distractor” words). The drift rates of old/studied and new/distractor words were summed to create an index of stimulus evaluation bias, which significantly increased post-exercise. A change in stimulus evaluation bias signifies a shift in the memory criterion, altering how the stimulus is processed and what evidence is extracted from the stimuli under consideration (White and Poldrack, 2014). An increase in stimulus evaluation bias (and thus, a more positive value) suggests all items seemed to provide more evidence as old/studied even if they were new/distractor stimuli. A more liberal memory criterion (i.e., require weaker memory/less evidence to identify word as “old/studied”) may be preferred when memory items are perceived as being harder to remember (Starns et al., 2012). Thus, individuals may have perceived the memory task as more difficult post-exercise and adapted a more lenient memory criterion, thereby inducing a stimulus evaluation bias and resulting in a tendency to identify all items as “old/studied” (increasing hits/false alarms). Whether the post-exercise changes in stimulus evaluation bias are a direct result of exercise or the experimental design itself is unclear and requires further scrutiny.

Significant reductions in post-exercise executive function (Flanker, trend for 2-back) and memory RT could stem from changes in encoding, the decision process itself, and motor execution. DDM was able to provide insight into the components of RT that are altered by acute exercise. We noted significant reductions in non-decision time during the 2-back and memory recognition tasks, with smaller effects trending toward statistical reductions during the Flanker task. As such, reductions in executive function and memory recognition RT observed herein were likely related to significant reductions in non-decision time (the sum of encoding and motor response phases that occur immediately prior to, and directly following, the actual decision making process). Previously, it was unclear if changes in RT post-exercise stemmed from alterations in stimulus evaluation and response selection (decision), or motor execution (Tsai et al., 2014). Neuroelectric proxies of stimulus evaluation duration (P3 latency; likely analogous to the decision component of DDM), may not be altered by exercise (Chu et al., 2015) although this is not universal (Kamijo et al., 2009). Our data indicate that improved executive function and memory processing speed post-exercise is largely independent from changes in the decision-making process itself (evidence strength, caution, bias) and is isolated to the encoding and motor response.

While we are unable to directly comment on whether exercise increases cognitive processing speed via accelerated visual encoding, motor response, or a combination of both, there is evidence that each may be altered post-exercise. Limited data suggest there are small changes in the time required for a motor response post-exercise (Ando et al., 2009) which could contribute to shorter non-decision time. It is unclear if accelerated encoding may explain the remaining reductions in post-exercise non-decision time. While, time required for visual encoding may decrease post-exercise owing to the residual effects of exercise on sensory cortex excitability (Bullock et al., 2017), some data suggest exercise does not alter neuroelectric indices of the initial sensory extraction from a cognitive stimulus (Chu et al., 2015). Additional contributors to post-exercise facilitation of non-decision time may include acute elevation of brain-derived neurotrophic factor and acute catecholamine/endorphin-mediated increases in arousal (McMorris et al., 2008; Dishman and O'Connor, 2009). Ultimately, the mechanisms behind the contributions of visual encoding and motor response to post-exercise changes in non-decision time, and in turn to RT, require further research.

### Implications

Our data suggest that brain plasticity, the characteristic that allows the brain to respond to a stimulus and improve processing speed (in this case, a single bout of exercise), is preserved in well-controlled, middle-aged HTN. Studying the neurocognitive response to a single bout of exercise is often viewed as a first step toward understanding how to favorably modulate resting functional state with habitual exercise training (Basso and Suzuki, 2017). In the present study, well-controlled non-obese HTN experienced similar beneficial increases in cognitive function following acute exercise as their non-HTN counterparts. A previous study from Teixeira et al. has noted no beneficial effects of a 12-week exercise program on cognitive function in resistant overweight/obese HTN (Teixeira et al., 2015). Additional research is needed to explore the relationship between acute exercise-cognitive responses and exercise training-cognitive adaptations in order to understand how to best use exercise as an adjuvant for improving cognitive health in HTN.

Prolonged sitting has been shown to have a detrimental effect on the brain, acutely reducing cognitive performance (Baker et al., 2018; Carter et al., 2018; Garcia-Hermoso et al., 2018). Time spent is sedentary pursuits may be slightly more detrimental to executive function in HTN adults compared to non-HTN adults (Steinberg et al., 2015). Replacing time spent in sedentary pursuits with physical activity/exercise has been shown to prevent acute declines in cognitive performance (Baker et al., 2018; Carter et al., 2018; Garcia-Hermoso et al., 2018) which may have implications for workplace productivity in non-HTN adults (von Thiele Schwarz and Hasson, 2011). Our findings may extend to HTN adults and suggest that if well-controlled, hypertension will not prevent the HTN adult from realizing the acute beneficial effects of exercise on cognitive function.

### Limitations

Our HTN group did not refrain from taking their anti-HTN medication during our study which may have influenced our findings. It is currently unclear if *acute* ingestion of anti-HTN medication directly impacts cognitive function. While acute doses of an ARB have been reported to enhance prospective memory in young normotensive adults (Mechaeil et al., 2011), this remains to be replicated in a clinically relevant population (i.e., hypertension). As such, future research should investigate the effects of acute anti-HTN medication on cognitive function following acute exercise. Whether responses are different among a group of older, or un-medicated HTN is additionally unclear and requires further research. We purposefully chose to interrogate the effect of exercise on cognitive function from 10 to 30 min post-exercise since we wished to identify if HTN individuals experienced the same benefits of exercise on cognitive function as their non-HTN counterparts and meta-analyses indicate this time period is sensitive to the acute effects of exercise (Chang et al., 2012). It is possible that further or differential changes in cognitive function could occur in these groups during prolonged recovery (i.e., >30 min) from a single bout of exercise. While this study was sufficiently powered to detect the larger effects of exercise on processing speed (RT), it may not have been adequate to detect subtle effects of both hypertension and exercise on processes of decision-making that might be identified with a larger sample. As such, larger studies utilizing similar novel methods may be required to reveal the potentially more subtle effects of hypertension and acute exercise on latent aspects of decision-making.

## Conclusions

We sought to investigate the effects of exercise on cognitive function in middle-aged HTN and non-HTN individuals and examine the effect of exercise on underlying processes of decision-making (via DDM). Both HTN and non-HTN individuals had similar cognitive responses following a single exercise bout of a recommended dose/intensity. While acute exercise did not alter task accuracy significantly, there were significant reductions in post-exercise RT for both executive function and memory domains in both groups. DDM revealed that changes in RT may largely stem from significant reductions in non-decision time which encompasses the early (visual encoding) and late (motor response) portions of the decision-making process.

## Author Contributions

WL, CW, and KH conceived and designed research. WL and JD collected and analyzed the data. All authors interpreted results. WL prepared figures and drafted manuscript. All authors edited, revised, and approved the final version of the manuscript.

### Conflict of Interest Statement

The authors declare that the research was conducted in the absence of any commercial or financial relationships that could be construed as a potential conflict of interest. The reviewer MN and handling Editor declared their shared affiliation and previous collaboration.
