# Supplementary material for: Effects of Acute Aerobic Exercise on Cognition and Constructs of Decision-Making in Adults With and Without Hypertension
Source: Front Aging Neurosci. 2019 Mar 8;11:41. doi: 10.3389/fnagi.2019.00041 (PMC6418781; doi:10.3389/fnagi.2019.00041)
Supplement: Supplementary file 1 [file Data_Sheet_1.docx]

| Supplemental Table S1: Reliability for executive function and memory recognition tasks. | | | | | |
| --- | --- | --- | --- | --- | --- |
|  | **Within-day**† | **Between-day (24-hr)**† | | **Between-day (1-7 d)** | **Within-day** |
|  | (n=19) | |  | (n=60) | |
|  | ICC | ICC |  | ICC | ICC |
| Flanker hits | **0.76** | **0.73** |  | **0.63** | **0.88** |
| Flanker RT | **0.74** | **0.87** |  | **0.81** | **0.92** |
| 2-back hits | 0.31 | 0.33 |  | **0.56** | **0.72** |
| 2-back RT | **0.82** | **0.84** |  | **0.59** | **0.85** |
| Memory hits | **0.62** | 0.29 |  | **0.68** | **-** |
| Memory RT | **0.80** | **0.69** |  | **0.66** | **-** |
| †separate, previous investigation. 2-back task did not require responses for non-match items. Bold denotes significant ICC (p<0.05).  ICC, intraclass correlation coefficient; RT, reaction time. | | | | | |
